# Enhanced stability of quantum Hall skyrmions under radio-frequency radiations

**DOI:** 10.1038/s41598-020-64505-3

**Published:** 2020-05-06

**Authors:** W. Pan, J. L. Reno, A. P. Reyes

**Affiliations:** 1 Sandia National Laboratories, Livermore, California USA; 2 Sandia National Laboratories, Albuquerque, New Mexico USA; 3 National High Magnetic Field Laboratory, Tallahassee, Florida USA

**Keywords:** Materials science, Nanoscience and technology

## Abstract

We present in this paper the results from a recent study on the stability of the quantum Hall skyrmions state at a Landau level filling factor (ν) close to ν = 1 in a narrow GaAs quantum well. Consistent with previous work, a resonant behavior is observed in the resistively detected NMR measurements. In the subsequent current-voltage (I-V) measurements to examine its breakdown behavior under radio frequency radiations, we observe that the critical current assumes the largest value right at the ^75^As nuclear resonant frequency. We discuss possible origin for this unexpectedly enhanced stability.

## Introduction

Not long ago, it was shown that a discrete time crystal^1–4^ can be realized if a quantum system is periodically driven to a non-equilibrium state. Proof-of-concept experiments are reported by two groups using trapped ions and nitrogen-vacancy centers in diamond, respectively^5,6^. The concept of discrete time crystals vividly demonstrates that the coherence time of a quantum system may be enhanced by driving the system out of equilibrium. In this paper, we want to test this novel concept in another canonical quantum system, the quantum Hall system in a two-dimensional electron gas (2DEG).

In a high quality 2DEG, discrete Landau levels are formed at low temperatures when a high magnetic field is applied perpendicularly. This gives rise to the integer quantum Hall effect (IQHE)^7^. In general, the IQHE can be understood under a single particle picture of Landau level quantization and disorder broadening^8^. However, this single-particle picture fails to explain the ν = 1 state. Here ν = *nh/eB* is the Landau level filling factor, *n* the density of 2DEG, *h* the Planck constant, *e* the electron charge, and *B* the magnetic field. At ν = 1, strong electron-electron (e-e) interactions and the Pauli principle force the electron spins to align with the external magnetic field, giving rise to a perfect quantum Hall ferromagnetic phase^9^. The lowest-energy charged excitations of this quantum Hall ferromagnetic phase are called skyrmions, a topological spin texture^9–11^. The quantum Hall skyrmions (QHS) state has been confirmed in various experiments^12–26^. Among the methods to probe the QHS state, the resistively detected nuclear magnetic resonance (RDNMR) technique^27^ is widely used. In a typical RDNMR measurement, an oscillatory radio frequency (RF) magnetic field is coupled to the, for example ^75^As, nuclear spins. The hyperfine interaction between the electron and nuclear spins, in turn, modifies the Zeeman energy and thus the resistance of the 2DEG at the Larmor frequency of ^75^As. As a result, a resonant behavior is observed in the spectrum of resistance versus RF frequency.

It is of great interest to ask the question about the stability of the QHS state when it is driven away from equilibrium. Answer to this question can provide a useful avenue to understanding quantum coherent properties of a non-equilibrium system. Indeed, compared to trapped ions and nitrogen-vacancy centers in diamond, the quantum Hall system can be realized in industrially compatible semiconductor materials and, thus, may have important implications in practical applications. A common approach to examine the stability of a quantum Hall (QH) system is to measure its energy gap^13–15^. Another commonly exploited method is to study its breakdown behavior^28–33^. In this kind of studies, a large current is applied to the quantum Hall specimen. As the current increases over a critical value, the QH effect breaks down and the resistance of 2DEG become non-zero. The size of the critical current is related to the stability of the QH system.

Here, we present the results from a recent study on the stability of the QHS state at a Landau level filling close to ν = 1 by measuring its current-voltage (I-V) breakdown characteristics under RF radiations. We observe that the critical current increases visibly when the RF frequency is right at the Larmor frequency of ^75^As nuclei, where the hyperfine interaction between electron and nuclear spins perturbs the QHS state most significantly. We believe that this observation is consistent with the novel concept that the coherence time of a quantum system may be enhanced by driving the system out of equilibrium.

## Device and Methods

The specimens used in our experiment are narrow GaAs quantum wells (QWs) sandwiched between two Al_0.24_Ga_0.76_As barrier layers of thickness 200 nm. The well width is 6 nm. The two-dimensional electron gas (2DEG) has a density of n ~ 4.5 × 10^10^ cm^−2^ and mobility of ~ 1 × 10^5^ cm^2^/Vs, after a brief red light-emitting diode (LED) illumination at low temperature (*T*). A standard low-frequency lock-in technique is used to measure the magneto-resistance R_xx_ and R_xy_ with an ac excitation current of 10 nA. Two quantum-well samples were studied, and the results are consistent with each other. In this article, we will present the results from one sample. For RDNMR measurements, an eight-turn pickup coil, made of copper wire, is wrapped around the specimen to couple the RF magnetic fields to the 2DEG.

## Results and Discussion

**Figure 1:**
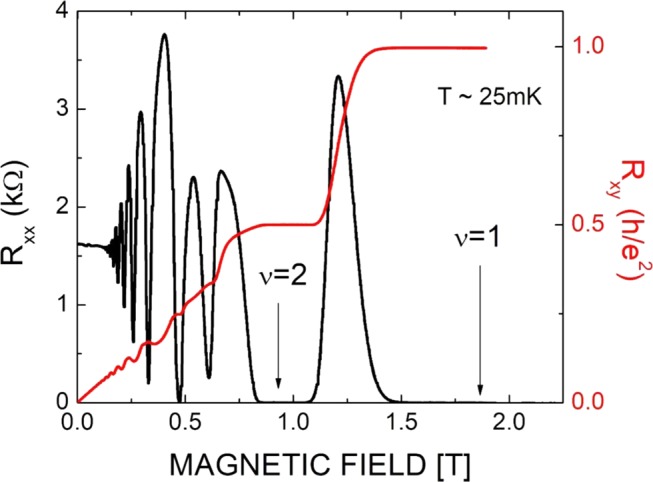
Shows the diagonal resistance R_xx_ (black curve) and Hall resistance R_x__y_ (red curve) of a narrow GaAs quantum well as a function of magnetic field at a temperature of T = 25 mK. Well developed quantum Hall effect is observed at Landau level filling ν = 1 and 2, marked by arrows.

Figure 1 shows the diagonal resistance R_xx_ and Hall resistance R_xy_ traces measured at *T* ~ 25 mK. At low magnetic fields, the Shubnikov-de Haas oscillations are clearly seen. In this regime, the spin degeneracy is not lifted and only the even Landau level filling states are developed. At higher magnetic fields, the spin degeneracy is lifted, and the developing ν = 3 state becomes visible. At even higher magnetic fields, well developed IQHE states are observed at ν = 1 and 2, R_xx_ is vanishingly small and R_xy_ is quantized to the expected values.

**Figure 2:**
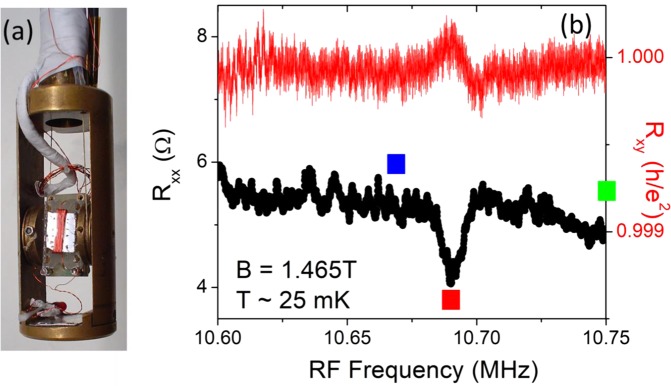
(**a**) Experimental setup for the resistively detected NMR. The specimen is mounted on a plastic chip carrier. An 8-turn coil (red colored) around the sample is used to couple RF radiations to the specimen. A red LED is used for low temperature illumination. (**b**) shows R_xx_ and R_xy_ as a function of RF frequency from 10.60 to 10.75 MHz. Resonant behavior is observed in both R_xx_ and R_xy_ at ~10.69 MHz. The blue, red, and green dots indicate the chosen frequencies under which the I-V measurements are performed, as shown in Fig. 3.

Next, we present results from our RDNMR measurements. Figure 2a shows a picture of the measurement setup. The shiny black piece on the chip-carrier is our specimen. The red NMR coil is wrapped around the middle of the specimen. Figure 2b shows the RDNMR results of R_xx_ and R_xy_ as a function of RF frequency (f). The magnetic field is fixed at *B* = 1.465 T (or the filling factor of ν = 0.85). f is swept from 10.6 to 10.75 MHz, at a rate of 10 kHz per step. R_xx_ is roughly constant but drops quickly at f = 10.69 MHz, before it recovers its constant value. This resonant behavior is consistent with the previous work^16–19,22,23,25^ on the quantum Hall skyrmions. Surprisingly, the R_xy_ trace also shows a resonant behavior at the same frequency but with a dispersive-like shape. Similar dispersive spectrum has also been observed in R_xx_ in previous studies^16,18,19,22,23,25^ and its origin remains unclear. Though the noise in the data prevents a quantitative comparison between the R_xy_ and R_xx_ spectra, nevertheless, by examining the shapes of R_xx_ and R_xy_ it is apparent that R_xx_ is proportional to -dR_xy_/df.

**Figure 3:**
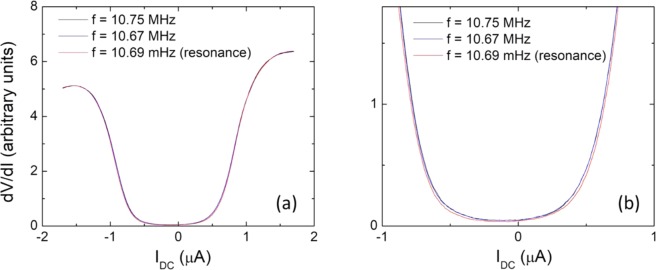
(**a**) Shows the dV/dI curves at the three selected RF frequencies of 10.67, 10.69, and 10.75 MHz. (**b**) the dV/dI curves zoomed in around the onset of breakdown points. It is clearly seen that the critical current (defined as the value at dV/dI = 1) is the largest at the resonance frequency of 10.69 MHz.

Having obtained the resonant behavior in our RDNMR measurements, we now show the results of I-V measurements under RF radiations, for the purpose of studying the stability of the quantum Hall skyrmions state. Three representative frequencies are chosen at f = 10.67 MHz, before the resonance, 10.69 MHz exactly at the resonant point, and 10.75 MHz away from the resonance (see Fig. 2b). In this kind of measurements, a d.c. current is added to the small a.c. bias current of δI = 10 nA and swept from −1.7 μA to 1.7 μA. A phase-sensitive lock-in amplifier is used to measure the a.c. voltage (δV) between two ohmic contacts. The obtained dV/dI is shown in Fig. 3a for all three frequencies. These curves display the typical quantum Hall breakdown behavior. At low DC current I_DC_, R_xx_ is small, close to zero. At a critical current close to ±1 μA, R_xx_ increases quickly, and then reaches a roughly constant value at higher currents. The slight asymmetry between the negative and positive currents is probably related to the edge states in the quantum Hall effect. Overall, the three curves are very similar and almost overlap each other. Yet, examining them closely in the region of the onset of breakdown, we notice that the critical current is slightly different for three curves. As shown in the zoomed plot of Fig. 3b, the two off-resonant curves overlap each other, while the on-resonant curve displays a larger critical current. To quantify the difference, we define the critical current (I_c_) at dV/dI = 1. Using this definition, we obtain I_c_^−^ = −0.789 μA and I_c_^+^ = 0.636 μA for f = 10.69 MHz; −0.780 and 0.623 μA for 10.67 MHz; and −0.775 and 0.623 μA for 10.75 MHz. Consequently, ΔI_c_ = I_c_^+^-I_c_^−^ = 1.425, 1.403, and 1.398 μA for f = 10.69, 10.67, and 10.75 MHz, respectively. Results are also listed in Table 1. It is obvious ΔI_c_ assumes the largest value right at the resonance frequency.

**Table 1:** The critical currents (in units of μA) at various RF frequencies (in units of MHz).

Critical Current	10.67 MHz	10.69 MHz	10.75 MHz
I_c_^+^ (μA)	0.623	0.636	0.623
I_c_^−^ (μA)	−0.780	−0.789	−0.775
ΔI_c_ (μA)	**1.403**	**1.425**	**1.398**

This observation is surprising. Right at the resonant point, the radio frequency magnetic fields, applied to the specimen through the pick-up coils, depolarize the ^75^As nuclear spins and cause them to precess. This, in turn, perturbs the electron spins through the change in the hyperfine interaction. In other words, at resonance condition, the RF magnetic fields should disturb the QHS state most strongly. As a result, a less stable QHS state and, thus, a smaller critical breakdown current are expected. To speculate the physical origin of the unexpected observed result, we mention that it has been shown that the coherence time, or stability, of a quantum system may be enhanced by periodically driving the system out of equilibrium. For example, a transient superconductivity has been achieved under an intense laser pulse^34^. It is possible that the enhanced stability of the quantum Hall skyrmions state in our quantum well specimen is due to the same mechanism. In general, due to the finite Coulomb energy of short spin waves and the small nuclear Zeeman energy, nuclear spin relaxation is hard to achieve even in the limit of vanishing electron Zeeman energy^35^. However, the gapless XY magnon mode of quantum Hall skyrmions system (probably as an overdamped mode in a skyrmions liquid state) can couple strongly to the nuclear spins because of its large S^x,y^ component and its gaplessness^36^. This coupling provides an efficient channel for spin transfer from the electrons to nuclei and vice versa. In our narrow quantum well, the Landé g-factor and, consequently, the ratio of Zeeman energy over Coulomb energy, are nearly zero. This favors the formation of large size skyrmions^17^. At the resonance frequency of 10.69 MHz, the nuclear spins polarization is driven out of their thermal equilibrium, and nuclear spins precess at their intrinsic Larmor frequency. Through coupling, this generates a periodically driving action on the gapless XY magnon mode and its S^x,y^ component. This scenario appears to resemble the formation of a time crystal for the case of the conserved component of the total moment in the XY plane^37^. Consequently, the resonant magnetic fields may help move our quantum Hall skyrmions system toward a many-body localization and, thus, stabilize the QHS state. As a result, the critical current becomes larger in the breakdown measurements.

## Conclusion

In summary, we have examined the stability of the quantum Hall skyrmions state at a Landau level filling close to ν = 1, by measuring its breakdown behavior under RF radiations. We observe that critical current where the quantum Hall skyrmions state breaks down is the highest at the resonant frequency obtained in the RDNMR measurements. We argue that this enhanced stability is consistent with the proposal that the coherence time of a quantum system may be enhanced by driving the system out of equilibrium.
